# Cytogenomic Integrative Network Analysis of the Critical Region Associated with Wolf-Hirschhorn Syndrome

**DOI:** 10.1155/2018/5436187

**Published:** 2018-03-12

**Authors:** Thiago Corrêa, Rafaella Mergener, Júlio César Loguercio Leite, Marcial Francis Galera, Lilia Maria de Azevedo Moreira, José Eduardo Vargas, Mariluce Riegel

**Affiliations:** ^1^Post-Graduate Program in Genetics and Molecular Biology, Universidade Federal do Rio Grande do Sul (UFRGS), 91501-970 Porto Alegre, RS, Brazil; ^2^Medical Genetics Service, Hospital de Clínicas de Porto Alegre, Rua Ramiro Barcelos 2350, 90035-903 Porto Alegre, RS, Brazil; ^3^Department of Pediatrics, Universidade Federal do Mato Grosso (UFMT), 78600-000 Cuiabá, MT, Brazil; ^4^Post-Graduate Program in Genetics and Biodiversity, Universidade Federal da Bahia, Campus Ondina, 40170-290 Salvador, BA, Brazil; ^5^Institute of Biological Sciences, Universidade de Passo Fundo, Passo Fundo, RS, Brazil

## Abstract

Deletions in the 4p16.3 region are associated with Wolf-Hirschhorn syndrome (WHS), a contiguous gene deletion syndrome involving variable size deletions. In this study, we perform a cytogenomic integrative analysis combining classical cytogenetic methods, fluorescence in situ hybridization (FISH), chromosomal microarray analysis (CMA), and systems biology strategies, to establish the cytogenomic profile involving the 4p16.3 critical region and suggest WHS-related intracellular cell signaling cascades. The cytogenetic and clinical patient profiles were evaluated. We characterized 12 terminal deletions, one interstitial deletion, two ring chromosomes, and one classical translocation 4;8. CMA allowed delineation of the deletions, which ranged from 3.7 to 25.6 Mb with breakpoints from 4p16.3 to 4p15.33. Furthermore, the smallest region of overlapping (SRO) encompassed seven genes in a terminal region of 330 kb in the 4p16.3 region, suggesting a region of susceptibility to convulsions and microcephaly. Therefore, molecular interaction networks and topological analysis were performed to understand these WHS-related symptoms. Our results suggest that specific cell signaling pathways including dopamine receptor, NAD+ nucleosidase activity, and fibroblast growth factor-activated receptor activity are associated with the diverse pathological WHS phenotypes and their symptoms. Additionally, we identified 29 hub-bottlenecks (H-B) nodes with a major role in WHS.

## 1. Introduction

Wolf-Hirschhorn syndrome (WHS, OMIM #194190) is a well known genetic condition with estimated prevalence of 1/20,000 to 1/50,000 births [1, 2] originally described independently by Hirschhorn and Cooper [3] and Wolf et al. [4] in the 1960s. The core clinical features of WHS are facial dysmorphia, growth retardation, intellectual disability, and seizures. Moreover, other clinical signs such as microcephaly, hypotonia, congenital heart defects, renal abnormalities, and skeletal anomalies have also been reported [5]. WHS is caused by a deletion in the p16.3 region (4p16.3), which has a variable size that reflects the spectrum and severity of the disease [5–7]. Approximately 55% of individuals with WHS exhibit the 4p16.3 deletion and approximately 40–45% are unbalanced translocations. Complex genomic rearrangements such as ring 4 chromosome may also be present in a smaller number of cases [6, 8].

The deleted region required to express the core WHS phenotype has been reported to encompass two adjacent critical regions. The first region described as the WHS critical region (WHSCR) encompasses a size of 165 kb interval on 4p16.3 [9]. Two genes are present in this region, the proximal part of WHSC1 and the negative elongation factor complex member A* (NELFA)*. WHSC1 is involved in controlling the expression of several genes and acts as an agent in chromatin remodeling [10]. The* NELFA* gene alters the expression of target genes by participating in regulating the elongation of RNA polymerase II transcription [11]. The second region is the WHSCR2, which encompasses the leucine zipper-EF-hand-containing transmembrane protein* (LETM)* gene and the 5′ end of WHSC1 [12, 13]. LETM1 encodes a protein involved in mitochondrial metabolism by transporting ions [14, 15]. Currently, the WHSCR is defined at chr4:419,224–2,010,962 position in the reference genome (NCBI BuildGRCh38/hg19) [16].

The possibility of using microarrays to characterize chromosomal rearrangements has led to several studies aimed at establishing genotype-phenotype correlations in WHS, and many of them have described the regions of susceptibility to seizures and microcephaly in patients with WHS [1, 5, 17–23]. However, no consensus has been reached on the exact identity of the genes and cell signaling pathways involved in promoting these symptoms. Therefore, we developed a cytogenomic integrative analysis that combines conventional cytogenetic techniques, chromosome microarray analysis (CMA), and systems biology strategies to suggest the mechanism underlying the seizures and microcephaly in WHS.

## 2. Materials and Methods

### 2.1. Study Design and Sample Selection

This was a retrospective study conducted on 16 samples from patients with clinical suspicions of WHS. The patients were followed up at the public genetic services in collaboration with the Brazilian Network of Reference and Information for Microdeletion Syndrome (RedeBRIM).

### 2.2. Cytogenetic Analysis

Karyotyping was performed on metaphase spreads prepared from peripheral blood samples. The chromosomal analysis was conducted after GTG banding at a 550-band resolution, and at least 100 cells from each patient were analyzed.

### 2.3. Fluorescence In Situ Hybridization (FISH)

The fluorescence in situ hybridization (FISH) experiments were carried out using standard techniques with commercially available locus-specific probes using a dual-color commercial probe for the WHSCR (Cytocell, UK). The probe for the 4p16.3 (red spectrum) contained a sequence that was homologous to the D4S166 locus and covered approximately 223 kb of this locus. The control probe for the 4q35.2 region (green spectrum) contained sequences that were homologous to the CTC-963K6 loci.

Hybridizations were analyzed using an epifluorescence microscope, and the images were captured using a charge-coupled device camera. At least 30 cells were analyzed per hybridization. We considered a chromosome region as deleted when the FISH signal from the corresponding probe was absent from one of the homologous chromosomes.

### 2.4. CMA

The deletions were mapped using whole-genome array-comparative genome hybridization (CGH) using a 60 mer oligonucleotide-based microarray with a theoretical resolution of 40 kb (8 × 60 K, Agilent Technologies Inc., Santa Clara, CA, USA). The labeling and hybridization were performed following the protocols provided by Agilent, 2011. The arrays were analyzed using a microarray scanner (G2600D) and the Feature Extraction software (version 9.5.1, both from Agilent Technologies). The images were analyzed using Cytogenomics v 2.0 and 2.7 with the statistical algorithm ADM-2 and a sensitivity threshold of 6.0.

### 2.5. Systems Biology Analysis

#### 2.5.1. Network Design

The Gene Multiple Association Network Integration Algorithm (GeneMANIA) version 3.1.2.8 (available at http://www.genemania.org/) was used to analyze the protein-protein interactions (PPI) networks based on the 343 genes obtained from the GENCODE V24–GRCh38/hg38-UCSC database. In the present study, the association data from GeneMANIA was based on the PPI databases, where each interaction between proteins is experimentally proven [24], or on gene coexpression data. The interactions based on known protein domains, pathways, and colocalization were not considered in the analysis because they could increase the false-positive ratios in PPI networks obtained. The outcomes obtained through these search engines were sequentially analyzed using Cytoscape 3.4.1 [25]. Nonconnected nodes from the networks were not included.

*(1) Clustering. *The MCODE tool was used to identify the densely connected and possibly overlapping regions in the Cytoscape network [26]. Dense regions corresponded to protein or compound-protein complexes or their parts. Then, Gene Ontology (GO) enrichment, Kyoto Encyclopedia of Genes and Genomes (KEGG), WikiPathways, and Reactome analyses were performed using the ClueGO Cytoscape plugin [27]. Based on the GO predictions, a cut-off of a* p*-corrected value ≤ 0.01 using the false discovery rate (FDR) algorithm (Bonferroni test) was used to describe the mechanisms as described in the discussion section.

*(2) Centralities. *Two major parameters of network centralities (degree and betweenness) were used to identify the hub-bottlenecks (H-B) nodes from the PPI network using the Cytoscape plugin CentiScaPe 3.2.1 [28]. The degree of centrality indicates the total number of adjacent nodes that are connected to a unique node. In this study, the average nodal degree of a network was defined as the sum of the different node degree scores divided by the total number of nodes that composed the entire network/s [29]. Furthermore, we also analyzed the betweenness, which corresponds to the number of shortest paths between two nodes that pass through a node of interest [28]. The arithmetic average of the betweenness parameter was estimated similarly to the average centrality degree [29].

### 2.6. Ethics Review

This study was approved by the Research Ethics Committee of the Hospital de Clínicas de Porto Alegre (HCPA) with approval number GPPG 10-560 and was conducted in accordance with all current institution ethical rules.

## 3. Results and Discussion

**Figure 1:**
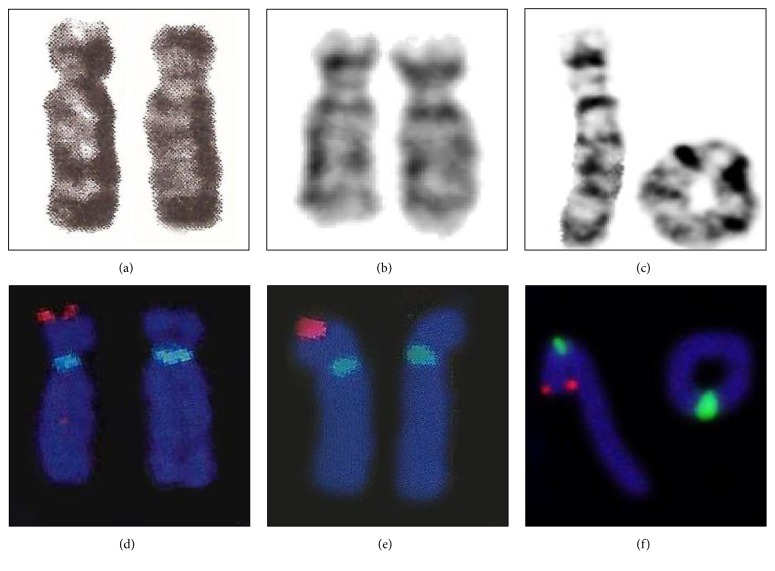
Karyotype results of (a) case 1 showing one normal chromosome 4 (left) and a chromosome 4p (right); (b) case 2, both normal chromosomes 4; (c) case 4, a normal chromosome 4 (left) and a ring chromosome 4 (right) and fluorescence in situ hybridization (FISH) results with locus-specific probes for the Wolf-Hirschhorn syndrome critical region (WHSCR) 4p16.3 from (d) case 1, (e) case 2, and (f) case 4. Absence of red signal on one copy of chromosome 4 indicates deletion of critical region.

**Figure 2:**
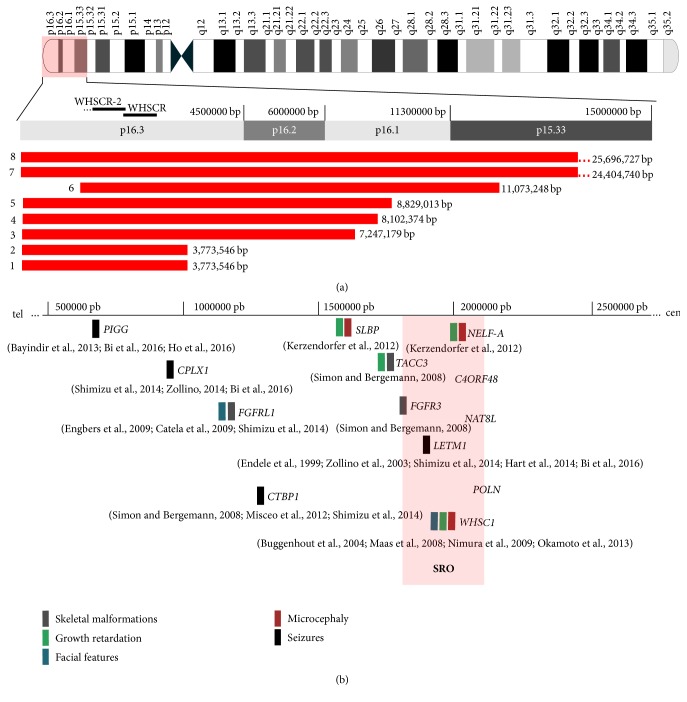
Cytogenomic profile of chromosome 4. (a) Red horizontal bars show extent of deleted segments on short arm of chromosome 4 in eight samples investigated using array- comparative genome hybridization (CGH). (b) Genes on 4p16.3p15.33 with haploinsufficiency effects associated with Wolf-Hirschhorn syndrome (WHS) clinical findings.

A total of 16 samples from patients whose clinical phenotypes were indicative of WHS were retrospectively evaluated (supplementary material S1). Among the patients, seven and nine (43.75 and 56.25%) were women and men, respectively. The most frequent clinical findings in our study group were seizures and microcephaly; however, facial dysmorphia, growth retardation, and intellectual disability were also observed (Table 1). In addition, the cytogenetic and FISH analysis identified 12 classical terminal deletions, one interstitial deletion, two ring chromosomes, and one translocation t(4;8) (p16.3;p23.1, Figure 1). Furthermore, from a total of 16 samples, the CMA was used to further map eight deletions that occurred caused by terminal or interstitial 4p16.3 rearrangements. The deletions ranged in size from 3.7 to 26 Mb (Figure 2(a)), and at least seven genes were within the smallest region of overlapping (SRO) deletion in the 4p16.3 region (Figure 2(b)). At the time of the present study there were no DNA samples available to perform CMA analysis from the further eight patients.

**Figure 3:**
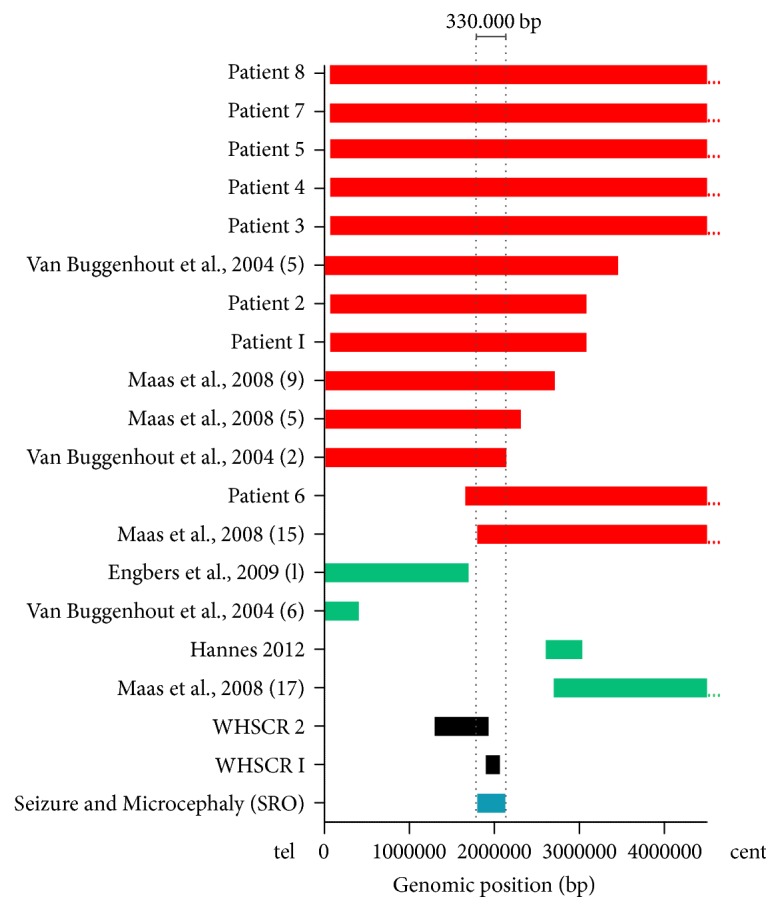
Smallest region of overlapping (SRO) associated with microcephaly and seizures. Bars show deletion sizes and genomic position on 4p. Red horizontal bars indicate seizures and microcephaly phenotype; green bars indicate absence of seizures and microcephaly, and two bars in gray represent critical regions of Wolf-Hirschhorn syndrome (WHS). The smallest region of susceptibility to microcephaly and seizures shown in this study is represented by blue bar, covering a 330 kb in size (1.8 to 2.13 Mb). WHSCR, Wolf-Hirschhorn syndrome critical region.

In our study, the SRO encompassed the 330 kb terminal region of the short arm of chromosome 4. This region extends from 1.8 to 2.13 Mb in the 4p16.3 region and it was identified as a region of susceptibility to the convulsions and microcephaly that are typical of WHS (Figure 2(b)). Comparing the cytogenomic profile of our samples with that of other studies, we delineated the proximal and distal breakpoints as the SRO [1, 17] in the 4p16.3 region (Figure 3). This region included seven candidate genes (*LETM1*,* FGFR3*,* WHSC1*,* NELFA*,* C4orf48*,* NAT8L*, and* POLN*). These genes could contribute to the pathogenic phenotype such as seizures and microcephaly and include the* LETM1* gene located in our SRO, which contributes to the presence of seizures in a hemizygous deletion [1, 12, 18, 30].

In human cell lines, the deletion of* LETM1* could alter the intracellular [Ca2+] levels, dysfunctional mitochondrial transition-pore opening, and hyperpolarization [31, 32]. Furthermore, these mitochondrial alterations could contribute to the emergence of some clinical findings in WHS including seizures [31, 32]. All patients in this study presented seizure symptoms, suggesting the putative involvement of this gene, although patients with 4p deletions, including* LETM1* without seizures, or patients with seizures and the presence of* LETM1* have been described [1, 17, 23, 33, 34]. However, this may be partially explained by the synergism between the phosphatidylinositol glycan anchor biosynthesis class G* (PIGG)*, complexin 1* (CPLX1)*, and* LETM1* genes, frequently associated with WHS convulsions [18].

Furthermore, our results showed a 330 kb region also associated with microcephaly. The deletion of two genes,* WHSC1* and* NELFA*, in this region has already been associated with this condition [17, 19, 35].* WHSC1* shows transcriptional corepressor activity by expressing a histone methyltransferase [32, 36], which controls the level of histone H3 lysine 36 (H3K36) trimethylation and histone acetylation [10].* NELFA* encodes a member of the negative elongation factor involved in regulating the progression of transcription by RNA polymerase II [37] and histone mRNA maturation into mRNAs [11]. The haploinsufficiency of* NELFA* in cell lines from patients with WHS is associated with delayed progression from the S- into the M-phase and altered chromatin assembly [35].

The following four additional genes are also located in the susceptibility region proposed in this study, fibroblast growth factor receptor 3* (FGFR3)*, N-acetyltransferase 8 like* (NAT8L)*, DNA polymerase Nu* (POLN)*, and chromosome 4 open reading frame 48* (C4ORF48I)*. However, these genes are not directly associated with seizures and microcephaly but have a putative involvement in the skeletal development and plasticity of the human brain [38–40]. In addition, previous studies have described the occurrence of seizures, microcephaly, or both, which indicates the contribution of multiple deleted genes located adjacent to the SRO delineated in our study [41].

**Figure 4:**
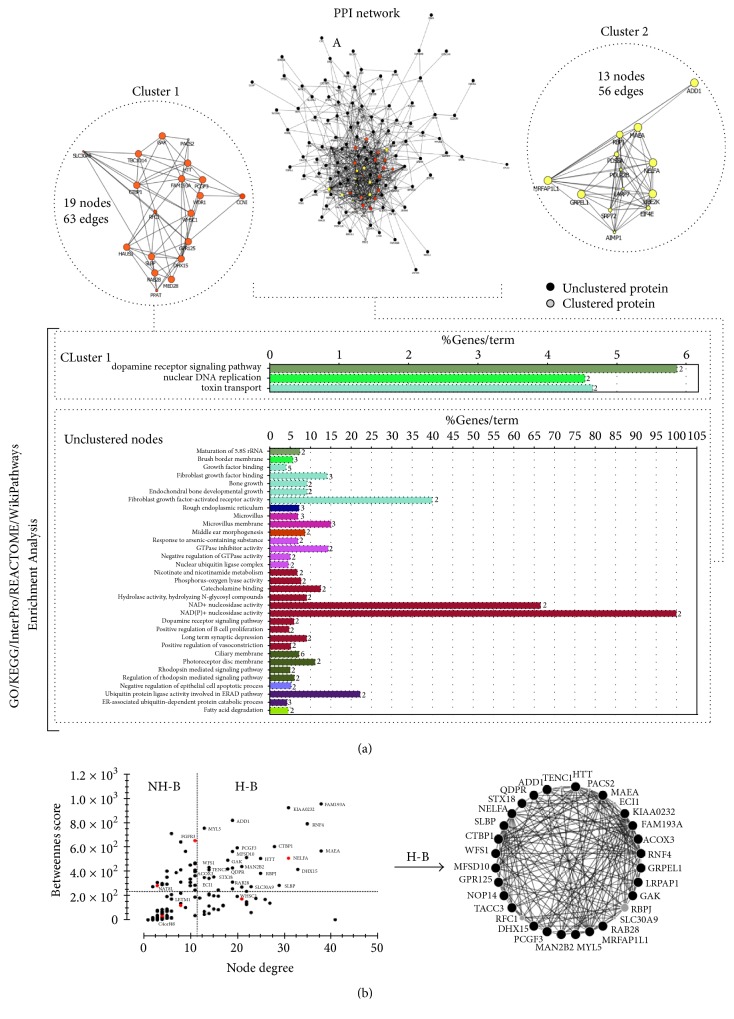
Graphs representing protein-protein interactions (PPI) network. (a) List of 343 genes was obtained from GENCODE V24-GRCh38/hg38-UCSC database. The data was used to construct networks using Cytoscape software processing. (b) Centralities parameters and topological analysis, using the CentiScaPe plugin; genes in small region overlapping (SRO) in our study are in red.

Thus, focusing on the critical 4p16.3 chromosome region mapped in our study, we explored the molecular interaction networks and biological pathways involving WHS-associated genes. An initial list of 343 genes obtained from the GENCODE V24-GRCh38/hg38-UCSC database was used to construct an interactome network. This network was composed of 136 nodes and 750 edges (Figure 4(a)). It is important to note that for this initial network prediction we considered a deletion of 26 Mb including our SRO, WHSCR, and WHSCR2 (Figure 2(a)). Furthermore, we performed a cluster analysis that identified two major cluster regions, cluster 1 (19 nodes and 56 edges) and cluster 2 (13 nodes and 56 edges). In these clustered and unclustered nodes, more significant GO categories were identified (Figure 4(a)). In the unclustered nodes, four discrete molecular functions were predicted: (1) NAD+ nucleosidase activity (*p* value corrected = 0.002); (2) NAD(P)+ nucleosidase activity (*p* value corrected = 7.5 × 10^−4^). These pathways participate in nicotinamide metabolism and calcium signaling, thereby contributing to excitability, exocytosis, motility, apoptosis, and cell transcription mechanisms [42, 43]; (3) FGFR activity (*p* value corrected = 0.006) and (4) FGF binding (*p* value corrected = 0.004), which is involved in the maintenance of tissue homeostasis and regulation of metabolic processes with specific roles such as the regulation of cell migration, proliferation, and differentiation [44, 45].

The receptors involved in these pathways are encoded by* FGFR3* and* FGFRL1*, which interact with FGFs to activate a downstream signaling cascade. The hemizygous deletions of* FGFR3* and* FGFRL1* are implicated in the skeletal abnormalities and facial features typical of WHS [21, 46–48]. Furthermore, three GO categories were identified in cluster 1 (Figure 4(a)). The dopamine receptor signaling pathway (*p* value corrected = 0.001) has been implicated in numerous neurological processes including sleep regulation, feeding, attention, cognitive functions, olfaction, and hormonal regulation [49]. Toxin transport (*p* value corrected = 0.001) and molecular functions such as nuclear DNA replication (*p* value corrected = 7.3 × 10^−4^) encompassing the stem-loop binding protein* (SLBP)* and* NELFA* genes, often deleted in WHS, were also predicted. It was not possible to predict GO and pathways categories for cluster 2.

An alternative strategy to decipher cell signaling pathways involved in seizures and microcephaly using networks is based on connectivity analysis. In this analysis, the centrality properties were evaluated (Figure 4(b)), and 29 hub-bottlenecks (H-B) nodes were identified in the initial network. The* NELFA* gene was localized in the SRO defined in this study, and the* SLBP* gene was identified as an H-B in the centrality analysis (Figure 4(b)). There is evidence that* NELFA* is necessary for the recruitment of* SLBP*, by its regulation of histone synthesis during the S-phase [11, 50]. Therefore, the haploinsufficiency of* NELFA*,* SLBP*, or both could affect the cell-cycle progression, DNA replication, and the chromatin assembly [35]. Interestingly,* NELFA* is located in the WHSCR and* SLBP* in WHCR2, suggesting the independent contribution of both genes in WHS pathogenesis.

The WHSC1 protein, also located in the SRO, affects the levels of trimethylated H3K36 (H3K36Me3), which can be reduced by siRNA-mediated knockdown of the NELF-E component of the NELF complex [10, 51]. This indicates another functional association between WHSC1 and NELFA in controlling gene expression by chromatin remodeling and regulation of the elongation of the transcription, respectively [35]. The identification of the H-B* NELFA*,* SLBP*, and the hub WHSC1 (Figure 4(b)) using centrality analysis highlights the importance of the involvement of these genes in the core WHS phenotype, such as skeletal malformation, facial features, and microcephaly (Figure 2(b)) [1, 17, 19, 35, 52].

The centrality analysis revealed that 90% and 10% of the H-B are located on chromosome 4 and other chromosomes, respectively. Considering this evidence, we suggest that the diversity of the pathological WHS phenotypes could be dependent on interrelated and close H-Bs located on the same chromosome. To the best of our knowledge, this is the first report of a cytogenomic integrative network analysis using systems biology to study the critical region associated with WHS. Our study described the putative cell signaling pathways altered in WHS that contribute to the integrative understanding of the role of contiguous genes in the spectrum of this syndrome.

## 4. Conclusions

This study combined clinical data and integrative analysis with conventional cytogenetic techniques, CMA, and systems biology strategies. We confined the region of susceptibility for microcephaly and seizures to a 330 kb sized region that encompassed seven candidate genes (*LETM1, FGFR3, WHSC1, NELFA, C4orf48, NAT8LI*, and* POLN*). The network topological analysis identified 29 H-Bs, and the candidate gene* NELFA* was included among these H-Bs. In addition, significant GO categories showed that several cell signaling pathways are responsible for the seizures and microcephaly in WHS.

## Figures and Tables

**Table 1:** Summary of cytogenomic and clinical findings of eight samples investigated using array-comparative genome hybridization (CGH).

	Case 1	Case 2	Case 3	Case 4	Case 5	Case 6	Case 7	Case 8	Total
Karyotype	46, XY, t(4:8)	46, XX	46, XX, 4p-	46, XX, r(4)	46, XX, 4p-	46, XY, 4p-	46, XY, 4p-	46, XY, 4p-	
*Molecular cytogenetic findings*									
FISH	del4p16.3	del4p16.3	del4p16.3	del4p16.3	del4p16.3	del4p16.3	del4p16.3	del4p16.3	
Deletion size (*pb*)	3,773,546	3,773,546	7,175,628	8,102,374	8,829,013	11,073,248	24,404,740	25,696,727	
Genomic position on chr:4 (GRCh38/hg38)	71552–3845097	71552–3845097	71552–7247179	71552–8173925	71552–8900564	1729442–12802689	68,345–24,473,084	68345–25765071	
*Clinical findings*									
Seizures	+	+	+	+	+	+	+	+	8/8
Microcephaly	+	+	+	+	+	+	+	+	8/8
Growth retardation	+	NA	NA	+	NA	+	+	+	5/8
Intellectual disability	+	NA	+	+	+	+	+	NA	6/8
Reserved prognosis	−	NA	−	+	+	−	+	−	3/8
Short upper lip	+	NA	+	+	+	+	+	+	7/8
Small mental region	+	NA	+	+	+	+	+	+	7/8
Labial deviations downward	+	NA	+	+	+	+	+	+	7/8
Hypoplastic columella	+	NA	+	+	+	+	+	+	7/8
Hypertelorism	+	NA	+	−	+	+	+	+	6/8
Ptosis of the eyelids	+	NA	+	−	+	+	−	NA	5/8
Deployment of hair on forehead is high	+	NA	+	−	+	+	+	+	6/8
Fine nose	+	NA	+	+	−	−	+	+	5/8
Cardiovascular malformations	NA	NA	+	−	−	−	+	−	2/8
Brain malformations	NA	NA	+	NA	−	−	+	−	2/8
Cleft palate	NA	NA	−	−	NA	NA	+	−	1/8
Renal malformations	NA	NA	−	−	−	NA	−	+	1/8
Hypospadias	NA	NA	−	NA	NA	NA	−	+	1/8
Feet and small hands	+	NA	+	+	+	+	+	−	6/8
Narrow fingers	+	NA	+	+	+	+	+	+	7/8
Hypotrophy of the thenar	+	NA	+	−	−	−	+	−	3/8
Hammer toe	NA	NA	+	−	−	−	−	−	1/8
Abnormal dermatoglyphs	−	NA	+	NA	NA	NA	NA	NA	1/8

(+) feature present; (−) feature absent; (NA) not available; FISH, fluorescence in situ hybridization.

## Supplementary Materials

Supplementary Materials S1: summary of cytogenomic and clinical findings of 16 samples from this study. Cytogenetic and clinical profiles from the retrospective study conducted on 16 samples from patients with clinical suspicions of WHS. Combining classical cytogenetic methods, fluorescence in situ hybridization (FISH), and chromosomal microarray analysis (CMA), we characterized 12 terminal deletions, one interstitial deletion, two ring chromosomes, and one classical translocation 4;8. CMA allowed delineation of the deletions in 8 samples, which ranged from 3.7 to 25.6 Mb with breakpoints from 4p16.3 to 4p15.33.
