# Supplementary material for: Cytogenomic Integrative Network Analysis of the Critical Region Associated with Wolf-Hirschhorn Syndrome
Source: Biomed Res Int. 2018 Mar 12;2018:5436187. doi: 10.1155/2018/5436187 (PMC5867687; doi:10.1155/2018/5436187)
Supplement: Supplementary Materials — S1: summary of cytogenomic and clinical findings of 16 samples from this study. Cytogenetic and clinical profiles from the retrospective study conducted on 16 samples from patients with clinical suspicions of WHS. Combining classical cytogenetic methods, fluorescence in situ hybridization (FISH), and chromosomal microarray analysis (CMA), we characterized 12 terminal deletions, one interstitial deletion, two ring chromosomes, and one classical translocation 4;8. CMA allowed delineation of the deletions in 8 samples, which ranged from 3.7 to 25.6 Mb with breakpoints from 4p16.3 to 4p15.33. [file 5436187.f1.docx]

S1: Summary of cytogenomic and clinical findings of 16 samples from this study.

|  | *Patient 1* | | *Patient 2* | | *Patient 3* | | *Patient 4* | | *Patient 5* | | *Patient 6* | | *Patient 7* | | *Patient 8* | | *Patient 9* | | *Patient 10* | | *Patient 11* | | *Patient 12* | | *Patient 13* | | *Patient 14* | | *Patient 15* | | *Patient 16* | | *Total* | |
| --- | --- | --- | --- | --- | --- | --- | --- | --- | --- | --- | --- | --- | --- | --- | --- | --- | --- | --- | --- | --- | --- | --- | --- | --- | --- | --- | --- | --- | --- | --- | --- | --- | --- | --- |
|  |  | |  | |  | |  | |  | |  | |  | |  | |  | |  | |  | |  | |  | |  | |  | |  | |  | |
|  |  | |  | |  | |  | |  | |  | |  | |  | |  | |  | |  | |  | |  | |  | |  | |  | |  | |
| Karyotype | 46, XY, t (4:8) | | 46, XX | | 46,XX, 4p- | | 46,XX,r(4) | | 46,XX, 4p- | | 46, XY,4p- | | 46, XY,4p- | | 46, XY,4p- | | 46, XX | | 46,XY,4p- | | 46, XY,4p- | | 46, XY,4p- | | 46,XX, 4p- | | 46,XY,r(4) | | 46, XY | | 46,XX, 4p- | |  | |
|  | |  | |  | |  | |  | |  | |  | |  | |  | |  | |  | |  | |  | |  | |  | |  | |  | |  |
|  | |  | |  | |  | |  | |  | |  | |  | |  | |  | |  | |  | |  | |  | |  | |  | |  | |  |
|  |  | |  | |  | |  | |  | |  | |  | |  | |  | |  | |  | |  | |  | |  | |  | |  | |  | |
| FISH | del4p16.3 | | del4p16.3 | | del4p16.3 | | del4p16.3 | | del4p16.3 | | del4p16.3 | | del4p16.3 | | del4p16.3 | | del4p16.3 | | del4p16.3 | | del4p16.3 | | del4p16.3 | | del4p16.3 | | del4p16.3 | | del4p16.3) | | del4p16.3 | |  | |
| Deletion size (pb) | 3,773,546 | | 3,773,546 | | 7,175,628 | | 8,102,374 | | 8,829,013 | | 11,073,248 | | 24,404,740 | | 25,696,727 | | NA | | NA | | NA | | NA | | NA | | NA | | NA | | NA | |  | |
| Deletion location on chr:4 (GRCh38/hg38) | 71552-3845097 | | 71552-3845097 | | 71552-7247179 | | 71552-8173925 | | 71552-8900564 | | 1729442-12802689 | | 68,345-24,473,084 | | 68345-25765071 | | ........ | | ........ | | …….. | | …….. | | …….. | | …….. | | …….. | | …….. | |  | |
|  |  | |  | |  | |  | |  | |  | |  | |  | |  | |  | |  | |  | |  | |  | |  | |  | |  | |
| *Clinical findings* |  | |  | |  | |  | |  | |  | |  | |  | |  | |  | |  | |  | |  | |  | |  | |  | |  | |
|  |  | |  | |  | |  | |  | |  | |  | |  | |  | |  | |  | |  | |  | |  | |  | |  | |  | |
| Seizures | + | | + | | + | | + | | + | | + | | + | | + | | + | | - | | + | | + | | NA | | + | | NA | | NA | | 12/16 | |
| Microcephaly | + | | + | | + | | + | | + | | + | | + | | + | | + | | NA | | NA | | NA | | + | | + | | NA | | NA | | 11/16 | |
| Growth retardation | + | | NA | | NA | | + | | NA | | + | | + | | + | | NA | | NA | | NA | | NA | | + | | + | | NA | | NA | | 7/16 | |
| Intellectual disability | + | | NA | | + | | + | | + | | + | | + | | NA | | + | | + | | + | | NA | | NA | | NA | | NA | | NA | | 9/16 | |
| Reserved prognosis | - | | NA | | - | | + | | + | | - | | + | | - | | NA | | NA | | NA | | NA | | NA | | NA | | NA | | NA | | 3/16 | |
| Short upper lip | + | | NA | | + | | + | | + | | + | | + | | + | | NA | | NA | | + | | NA | | + | | NA | | NA | | NA | | 9/16 | |
| Small mental region | + | | NA | | + | | + | | + | | + | | + | | + | | NA | | NA | | NA | | NA | | + | | NA | | NA | | NA | | 8/16 | |
| Labial deviations downward | + | | NA | | + | | + | | + | | + | | + | | + | | NA | | NA | | + | | NA | | + | | NA | | NA | | NA | | 9/16 | |
| Hypoplastic columella | + | | NA | | + | | + | | + | | + | | + | | + | | NA | | NA | | NA | | + | | - | | NA | | NA | | NA | | 8/16 | |
| Hypertelorism | + | | NA | | + | | - | | + | | + | | + | | + | | + | | + | | + | | NA | | + | | NA | | NA | | NA | | 10/16 | |
| Ptosis of the eyelids | + | | NA | | + | | - | | + | | + | | - | | NA | | NA | | NA | | NA | | NA | | + | | NA | | NA | | NA | | 5/16 | |
| Deployment of hair on forehead is high | + | | NA | | + | | - | | + | | + | | + | | + | | NA | | NA | | NA | | NA | | NA | | NA | | NA | | NA | | 6/16 | |
| Fine nose | + | | NA | | + | | + | | - | | - | | + | | + | | NA | | NA | | NA | | NA | | - | | NA | | NA | | NA | | 5/16 | |
| Cardiovascular malformations | NA | | NA | | + | | - | | - | | - | | + | | - | | NA | | + | | + | | + | | + | | NA | | NA | | NA | | 6/16 | |
| Brain malformations | NA | | NA | | + | | NA | | - | | - | | + | | - | | NA | | NA | | NA | | + | | - | | NA | | NA | | NA | | 3/16 | |
| Cleft palate | NA | | NA | | - | | - | | NA | | NA | | + | | - | | NA | | NA | | NA | | NA | | + | | NA | | NA | | NA | | 2/16 | |
| Renal malformations | NA | | NA | | - | | - | | - | | NA | | - | | + | | + | | + | | NA | | NA | | + | | NA | | NA | | NA | | 4/16 | |
| Hypospadias | NA | | NA | | - | | NA | | NA | | NA | | - | | + | | NA | | + | | + | | + | | - | | NA | | NA | | NA | | 4/16 | |
| Feet and small hands | + | | NA | | + | | + | | + | | + | | + | | - | | NA | | NA | | NA | | NA | | + | | NA | | NA | | NA | | 7/16 | |
| Narrow fingers | + | | NA | | + | | + | | + | | + | | + | | + | | NA | | NA | | NA | | NA | | - | | NA | | NA | | NA | | 7/16 | |
| Hypotrophy of the thenar | + | | NA | | + | | - | | - | | - | | + | | - | | NA | | NA | | NA | | NA | | + | | NA | | NA | | NA | | 4/16 | |
| Hammer toe | NA | | NA | | + | | - | | - | | - | | - | | - | | + | | NA | | NA | | NA | | - | | NA | | NA | | NA | | 2/16 | |
| Abnormal dermatoglyphs | - | | NA | | + | | NA | | NA | | NA | | NA | | NA | | NA | | NA | | + | | NA | | NA | | NA | | NA | | NA | | 2/16 | |

(+) feature present; (-) feature absent; (NA) not available.
